# Structural Elements Regulating Amyloidogenesis: A Cholinesterase Model System

**DOI:** 10.1371/journal.pone.0001834

**Published:** 2008-03-19

**Authors:** Létitia Jean, Chiu Fan Lee, Michael Shaw, David J. Vaux

**Affiliations:** 1 Sir William Dunn School of Pathology, University of Oxford, Oxford, United Kingdom; 2 Clarendon Laboratory, Department of Physics, University of Oxford, Oxford, United Kingdom; Fred Hutchinson Cancer Research Center, United States of America

## Abstract

Polymerization into amyloid fibrils is a crucial step in the pathogenesis of neurodegenerative syndromes. Amyloid assembly is governed by properties of the sequence backbone and specific side-chain interactions, since fibrils from unrelated sequences possess similar structures and morphologies. Therefore, characterization of the structural determinants driving amyloid aggregation is of fundamental importance. We investigated the forces involved in the amyloid assembly of a model peptide derived from the oligomerization domain of acetylcholinesterase (AChE), AChE_586-599_, through the effect of single point mutations on β-sheet propensity, conformation, fibrilization, surfactant activity, oligomerization and fibril morphology. AChE_586-599_ was chosen due to its fibrilization tractability and AChE involvement in Alzheimer's disease. The results revealed how specific regions and residues can control AChE_586-599_ assembly. Hydrophobic and/or aromatic residues were crucial for maintaining a high β-strand propensity, for the conformational transition to β-sheet, and for the first stage of aggregation. We also demonstrated that positively charged side-chains might be involved in electrostatic interactions, which could control the transition to β-sheet, the oligomerization and assembly stability. Further interactions were also found to participate in the assembly. We showed that some residues were important for AChE_586-599_ surfactant activity and that amyloid assembly might preferentially occur at an air-water interface. Consistently with the experimental observations and assembly models for other amyloid systems, we propose a model for AChE_586-599_ assembly in which a steric-zipper formed through specific interactions (hydrophobic, electrostatic, cation-π, SH-aromatic, metal chelation and polar-polar) would maintain the β-sheets together. We also propose that the stacking between the strands in the β-sheets along the fiber axis could be stabilized through π-π interactions and metal chelation. The dissection of the specific molecular recognition driving AChE_586-599_ amyloid assembly has provided further knowledge on such poorly understood and complicated process, which could be applied to protein folding and the targeting of amyloid diseases.

## Introduction

Protein misfolding can be deleterious by triggering aggregation and insolubilization. In turn, the aggregation can lead to toxic conformation during which polymerization by folding and stacking of cross-β sheets result in the formation of amyloid fibrils. Fibril formation is a multiple kinetic event during which an energetically unfavourable nucleated polymerization (characterized by a lag phase) initiates the formation of a minimal self-assembled complex (nucleus or seed) serving as a structural template for a cooperative amyloid elongation [1]. Amyloid fibrilization is proposed to be the molecular basis of and the common link between a variety of pathological conditions and human neurodegenerative syndromes, such as type II diabetes, Alzheimer's, Parkinson's, Huntington's and prion diseases [2]. Although amyloid formation and deposition is a common feature of these diseases, the amyloid fibrils originate from different and distinct proteins or peptides that do not appear to share any sequence homology or function. However, fibrils formed from these amyloid-related sequences possess similar structural, physical and chemical properties, including formation of β-sheets whose strands run perpendicular to the fibril axis, fibril morphology and typical X-ray diffraction pattern, kinetic pattern of fibril formation and staining with dyes such as Congo red and Thioflavin T (ThT) [3], [4], [1], [5], [6]. Therefore, amyloid formation involves more than non-specific aggregation and non-specific hydrophobic interactions and it is recognized that some levels of structural complexity and specific pattern of interactions are important [7], [8], [9], [10]. Indeed, certain types of residues characterized by high β-sheet propensity, and/or fastest kinetics of aggregation, and/or stabilizing and assembling properties have been found to be commonly present in amyloid-related sequences [11], [12], [13], [14], [15]. Consequently in recent years, a great deal of attention has focussed on determining the factors and interactions resulting in fibrilization and finding common rules that govern the assembly. Such detailed dissections of the specific molecular recognition and self-assembly during amyloid formation could provide invaluable knowledge for the targeting and control of diseases involving toxic protein aggregation and deposition.

During Alzheimer's disease (AD) pathogenesis, the accumulation in the brain of extracellular amyloid-β-peptide (Aβ) in senile plaques and of intracellular hyperphosphorylated Tau in neurofibrillary tangles are thought to represent the hallmarks of the disease [16]. However, other proteins have also been implicated in the pathology, with one example being acetylcholinesterase (AChE) [17]. AChE is associated with senile plaques, promotes Aβ fibrilisation, and triggers early disease and increases plaque burden in double transgenic mice expressing human amyloid precursor protein (hAPP, from which Aβ is proteolytically cleaved) and hAChE when compared to single transgenic hAPP mice [18], [19], [20]. We have studied a 14 residue peptide named AChE_586-599_, which corresponds to a region within the C-terminal oligomerization domain of human AChE. The region encompassing AChE_586-599_ shares homology with Aβ and possesses high propensity for conversion to non-native (hidden) β-strand, a property associated with amyloidogenicity [21], [22]. Moreover, AChE_586-599_ adopts a β-sheet conformation, self-assembles into amyloid fibrils and promotes Aβ fibrilization [23], [24]. This peptide represents a tractable model for studying amyloid formation because its fibrilization is highly dependent upon pH. This allows a total control of the start of the polymerization process, which is triggered by the addition of physiological buffer to an acid solution. Moreover, AChE_586-599_ is an attractive model due to its residue composition with alternating charged, polar and hydrophobic amino acids most of which have been previously shown to be implicated in amyloid formation [11], [12], [15], [25]. Understanding how AChE_586-599_ residue composition and chemical nature affect the polymerization process should provide insights and strengthen current knowledge into complex networks of interactions leading to amyloid fibril formation.

In this study, we examined the role of each residue within AChE_586-599_ through the effect of single point mutations on the β-sheet propensity, conformation, fibrilization, surfactant activity, oligomer formation and fibril morphology of AChE_586-599_. We determined the importance of residues and the potential molecular interactions underlying AChE_586-599_ assembly into β-sheets and during the stacking of these sheets. Non-covalent side chain-side chain interactions, such as hydrophobic, cation-π and π-π interactions, were found to be critical for fibrilization and assembly stabilization. This detailed analysis allowed us to propose a model for the amyloid polymerization of AChE_586-599_ in which specific interactions between residue side-chains lead to the formation of a steric-zipper maintaining the β-sheets together, and π-π interactions allow the stacking and arrangement of strands within a β-sheet.

## Results

To determine the role of each residue of AChE_586-599_ in the process of amyloid formation, a library of alanine scanning mutants along with a structurally conserved substitution mutant (Tyr to Phe) and a truncation mutant (missing the last residue) were used.

### Identification of the residues important for the β-sheet propensity of AChE_586-599_ and its conformational transition from random coil to β-sheet upon neutralization

**Figure 1:**
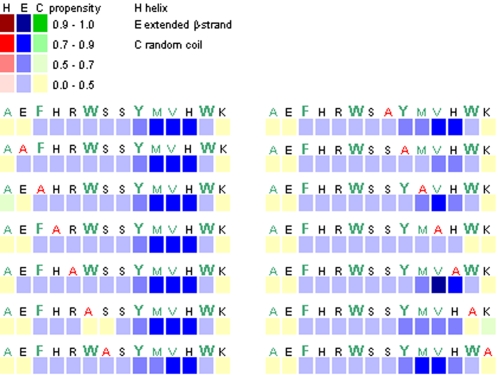
Secondary structure propensity of AChE_586-599_ mutants as predicted by hidden β-propensity method (available at http://opal.umdnj.edu). Propensities for helices (red squares), β-strands (blue squares) and random coil (green squares) are presented numerically using a 0-1 scale, with low values indicating zero to low propensity and high values indicating high propensity to near certainty. Hydrophobic residues are shown in green and aromatic residues in bold with a bigger font size.

We performed secondary structure prediction in term of high propensity for conversion to non-native (hidden) β-strand, using the method described by Yoon and Welsh [22]. Previously, Yoon and Welsh have predicted the minimal amyloidogenic regions for Aβ and α-synuclein, and have also identified AChE_586-599_ to be a region of AChE with high non-native (hidden) β-strand propensity [22]. Since the amyloidogenicity of a peptide has been associated with its β-sheet forming propensity, such analysis could provide an insight on the importance of certain residues in the fibrilogenicity of AChE_586-599_. When we applied the algorithm to AChE_586-599_, the whole peptide (with the exceptions of the N-terminal Ala and Glu, and the C-terminal Lys) possessed propensity for conversion to β-strand with the strongest propensity for the sequence YMVH (Figure 1). Some mutations (E_2_ and R_5_) increased the β-strand propensity. The H_12_/A mutant strengthened the propensity for V_11_ albeit decreasing it slightly for Y_9_ and M_10_. By contrast, some mutations drastically impaired the β-strand propensity in some part of the sequence: W_6_, Y_9_, M_10_, V_11_ and W_13_. The W_6_/A mutant created a break in the continuity of β-strand propensity, whereas the Y_9_/A, M_10_/A, V_11_/A and W_13_/A mutants drastically decreased it in the YMVH region. Four other mutations also impaired the β-strand propensity but moderately: F_3_ triggering a stronger random coil propensity for A_1_; S_7_, S_8_ and K_14_ slightly decreasing the β-strand propensity in the YMVH region. Only the mutation to Ala did not affect the β-strand propensity. Thus, W_6_, Y_9_, M_10_, V_11_ and W_13_ appeared to be crucial for maintaining a high β-strand propensity along the entire sequence.

**Figure 2:**
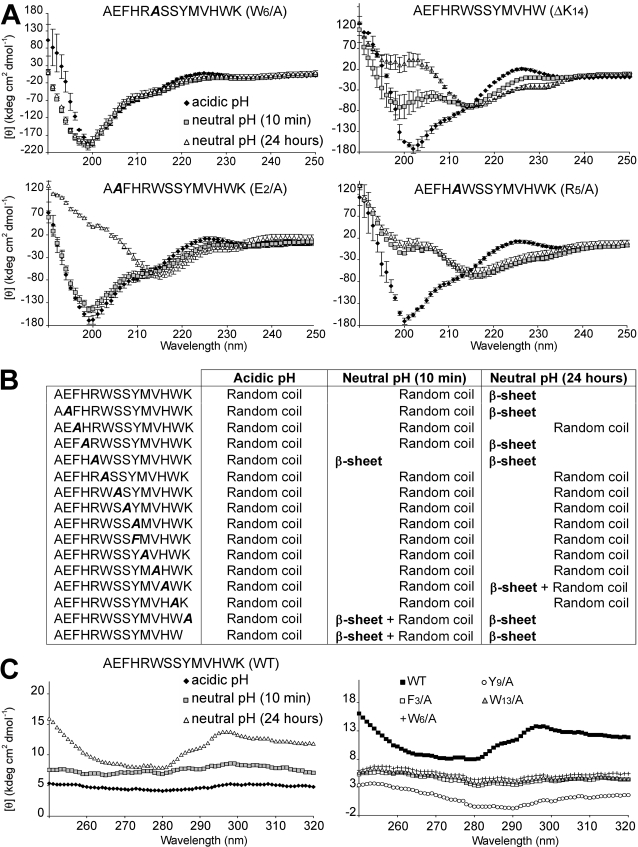
Conformation of AChE_586-599_ and AChE_586-599_ mutants. (A) Far UV spectra (250 to 190 nm) before and after pH neutralization (50 mM NaH_2_PO_4_, pH 7.2) of 4 AChE_586-599_ mutants (100 µM). These spectra are representatives of the different structures and different changes in structure observed for the wild-type and mutant peptides. (B) Conformation and changes in conformation after pH neutralization (50 mM NaH_2_PO_4_, pH 7.2) for AChE_586-599_ and all AChE_586-599_ mutants (100 µM). (C) Near UV spectra (320 to 240 nm) before and after pH neutralization (50 mM NaH_2_PO_4_, pH 7.2) of AChE_586-599_ and 4 mutants (100 µM). In all panels, the mutation within AChE_586-599_ is indicated in bold and italics.

Far-UV circular dichroism (CD) studies were performed to establish the conformation of AChE_586-599_ and AChE_586-599_ mutants before and after neutralization, allowing us to follow early conformational changes that preceded and occurred during aggregation. AChE_586-599_ was previously shown to be random coil when non-aggregated and to switch to a β-sheet structure upon neutralization [23]. Representatives of the conformations and changes in conformation observed are presented in Figure 2A and the conformations found for all the peptides at the different pHs are summarized in Figure 2B. CD spectra typical for a random coil structure (negative molar ellipticity at 200 nm or below) were observed for all peptides at acidic pH, indicating their non-aggregated status under acidic conditions. Nine mutant peptides still displayed random coil spectra after neutralization (e.g. W_6_/A, top left panel in Figure 2A) (Figure 2B). Therefore, these mutants were not able to adopt a β-sheet conformation under the conditions of the assay, which suggested the importance of the hydrophobicity and/or aromaticity of F_3_, W_6_, S_7_, S_8_, Y_9_, M_10_, V_11_ and W_13_ in the conformational transition upon neutralization. By contrast, negative molar ellipticity around 215 nm was found at neutral pH for 6 peptides: AChE_586-599_, E_2_/A, H_4_/A, R_5_/A, K_14_/A and ΔK_14_ mutants (Figure 2A, bottom panels, and Figure 2B). Such a negative ellipticity is typically assigned to β-sheet structures. Some peptides started to adopt a partial (e.g. ΔK_14_ mutant, Figure 2A, top right panel with double negative ellipticities at 200 and 215 nm) or a complete β-sheet structure (e.g. R_5_/A mutant, Figure 2A bottom right panel) after only 10 min at neutral pH. These results indicated that such mutants were faster than AChE_586-599_ at adopting a β-sheet conformation, and therefore that the Arg and the Lys residues are not crucially involved in the conformational change during neutralization and may even have a negative effect. After 24 hours at neutral pH, visual inspection of the solutions for the peptides adopting a β-sheet structure revealed the presence of insoluble aggregates, indicating tertiary or quaternary arrangements possibly leading to the formation of intermolecular-stacked β-sheets (see below).

We then applied near-UV CD to follow the behavior of the aromatic side-chains during aggregation. AChE_586-599_ does not possess Cys residues, therefore any bands in the near-UV spectrum can only be attributed to constraints on the side-chains of aromatic residues. Before neutralization, the AChE_586-599_ spectrum did not exhibit any strong positive bands, indicating the absence of conformational restriction of the aromatic side-chains (Figure 2C, left panel). By contrast, the near-UV spectrum of AChE_586-599_ after 24 hours at neutral pH showed three positive bands (below 260 nm, at 287 nm and 297 nm), which were consistent with conformational constraints on the aromatic rings of Phe and Tyr and/or Trp. By contrast the spectra of mutants, in which a single aromatic residue was substituted to Ala (the F_3_/A, W_6_/A, Y_9_/A and W_13_/A mutants), did not display any positive bands (Figure 2C, right panel). Therefore, the formation of the insoluble aggregates of AChE_586-599_ was associated with tertiary or quaternary interactions involving aromatic residues.

### Identification of the residues important for the fibrilization properties of AChE_586-599_

**Figure 3:**
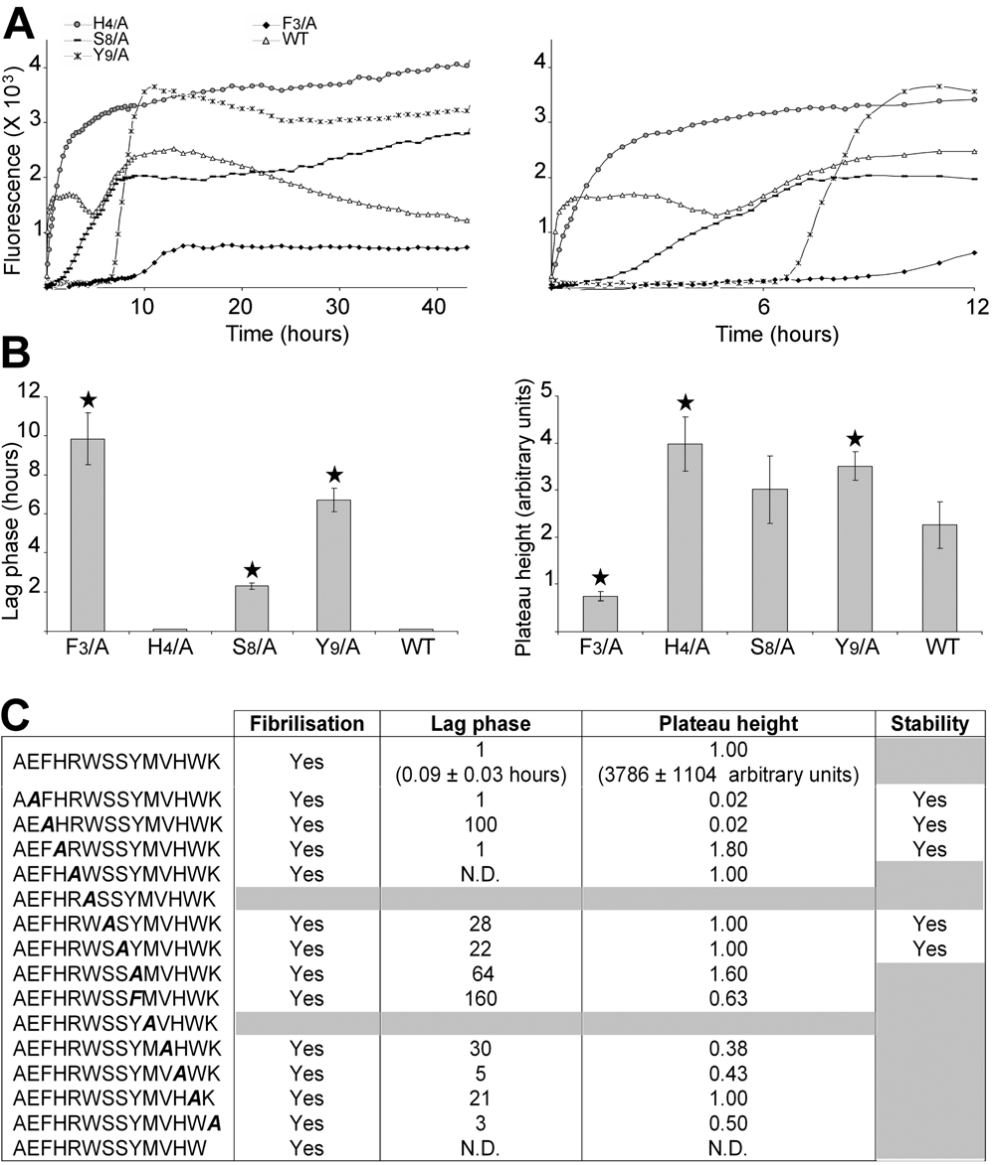
Fibrilization properties of AChE_586-599_ and AChE_586-599_ mutants. (A) 100 µM peptide was incubated with 165 µM ThT in PBS. Changes in ThT fluorescence were monitored (A, with the right panel showing scale-up of the left panel to visualize the rapid fibrilization of some peptides) with the lag phase of fibrilization (B, left panel) and plateau height (B, right panel) depicted. A black star signifies p<0.003 (B, left panel) and p<0.03 (B, right panel) when compared to the wild type peptide (WT). The peptides shown are representatives of the different fibrilization properties observed. (C) Fibrilization properties for AChE_586-599_ and all AChE_586-599_ mutants (100 µM). The properties are divided into 4 categories: ability to fibrilize, duration of lag phase, height of plateau and stability of the amyloid products (indicated by stability or decay of the ThT fluorescence after plateau). The mutation within AChE_586-599_ is indicated in bold and italics. The peptides that do not fibrilize and/or the peptides which amyloid products are not stable are indicated by grey boxes. The lag phase and plateau height for the mutant peptides are shown as fold ratio of AChE_586-599_ (e.g. ‘1’ represents equal value to AChE_586-599_ and ‘100’ for the lag phase represents 100 times longer than AChE_586-599_). ‘N.D.’ means ‘not detectable’.

The ability to form amyloid and the fibrilization kinetics of AChE_586-599_ and AChE_586-599_ mutants were determined by changes in ThT fluorescence emission in shaking conditions. Shaking was used to accelerate fibrilization, which was necessary at least for some mutants. After a lag phase of 0.09 hours, AChE_586-599_ rapidly self-assembled into amyloid aggregates (Figure 3A). It has to be noted that the kinetics of AChE_586-599_ assembly involved 2 phases, with a first rapid assembly (<1 hour) followed by a short plateau and decrease of ThT signal, and a second assembly with a slower rate (between 4.6 and 12 hours) also followed by a short plateau and decrease of ThT signal (Figure 3 A). However, AChE_586-599_ assembly performed in quiescent conditions did not display this biphasic behavior (data not shown). Instead, it revealed that the assembly started immediately, without the first assembly, but instead resembling the second assembly observed during the shaking conditions. This assembly in quiescent conditions was also followed by a short plateau and a decrease of ThT signal identical to the ones observed during shaking conditions. Therefore, AChE_586-599_ biphasic behavior was triggered by the shaking environment, which might either affect the assembly susceptibility to breakage by increasing shear forces, or affect the peptide surfactant activity by increasing the surface area and peptide recruitment. Collectively, the decay of the ThT signal after plateau in both conditions (shaking or not) suggested that the assembly of AChE_586-599_ was not stable.

Most of the substitutions affected both the lag phase and plateau height (Figure 3A, B and C). The exceptions were the E_2_/A and H_4_/A mutants, which showed a similar lag phase to AChE_586-599_, however their plateau height was affected with a drastic decrease for the E_2_/A mutant and an increase for the H_4_/A mutant. For both mutants, the substitution triggered stability of the assembly. The R_5_/A, S_7_/A, S_8_/A and W_13_/A mutants showed a similar plateau height to AChE_586-599_. However, their lag phases were increased, except for the R_5_/A mutant.

Substitution of H_12_ and K_14_ reduced the kinetics of fibrilization but did not abolish it completely. Indeed, slight increases of the lag phase were observed (5 and 3 times longer than AChE_586-599_, respectively), however their plateau heights were reduced on average by half (Figure 3C). Aggregation of the R_5_/A and ΔK_14_ mutants exhibited a significantly shorter lag phase than AChE_586-599_, which indicated a lower kinetic solubility and higher kinetic rate of fibrilization (Figure 3C). In fact, the R_5_/A and ΔK_14_ mutants immediately aggregated. These results suggested that positive charges from the side-chains of R_5_ and K_14_ might induce repulsion not favorable to a rapid aggregation and therefore might control assembly through specific interactions. By contrast, substitution of F_3_ to Ala and Y_9_ to Ala or Phe resulted in a dramatically decreased aggregation rate, with a very long lag phase (respectively 100, 64 or 160 times slower than AChE_586-599_) and a lower plateau height (except for the Y_9_/A mutant) (Figure 3A and B). The W_13_/A mutant was also affected with a lag phase 21 times slower than AChE_586-599_ (Figure 3C). These results suggested the importance of aromatic residues in the first stage of aggregation of AChE_586-599_. Interestingly, the substitution of Y_9_ to Phe affected the rate of aggregation even more than the substitution to Ala (Figure 3C). The most dramatic effect was that of the substitution of W_6_ and M_10_ to Ala, leading to a total loss of the peptide ability to form any aggregates as far as could be detected in the assay (Figure 3C). Finally, the substitutions to Ala of E_2_, F_3_, H_4_, S_7_ and S_8_ triggered the AChE_586-599_ assembly to be stable (Figure 3A and C).

Some mutants had very low level of fibrilization (very low plateau height) (e.g. the F_3_/A mutant), which may explain the absence of β-sheet conformation by CD for these mutants. Furthermore, the ThT assay done in similar conditions to the CD (no shaking, 24 hours) led to very poor fibrilization (long lag phase and low plateau height) or absence of fibrilization for the S_7_/A, S_8_/A, Y_9_/A and W_13_/A mutants (data not shown), which would also explain the absence of β-sheet conformation by CD.

### Effect of electrostatic interactions and ionic strength on the stability of AChE_586-599_ aggregates

To examine the effect of charge neutralization on AChE_586-599_ fibril formation, we used increasing salt concentrations (NaCl and KCl) in a ThT assay, and the effects on both the E_2_/A and K_14_/A mutants were analysed. The strategy for selecting these mutants was based upon the presence of opposite charges on their side-chain at neutral pH, their position at the AChE_586-599_ termini and their fibrilization properties (see above). Moreover, removing the side-chain charge from one of them was potentially leaving the side-chain charge from the other one uncompensated.

**Figure 4:**
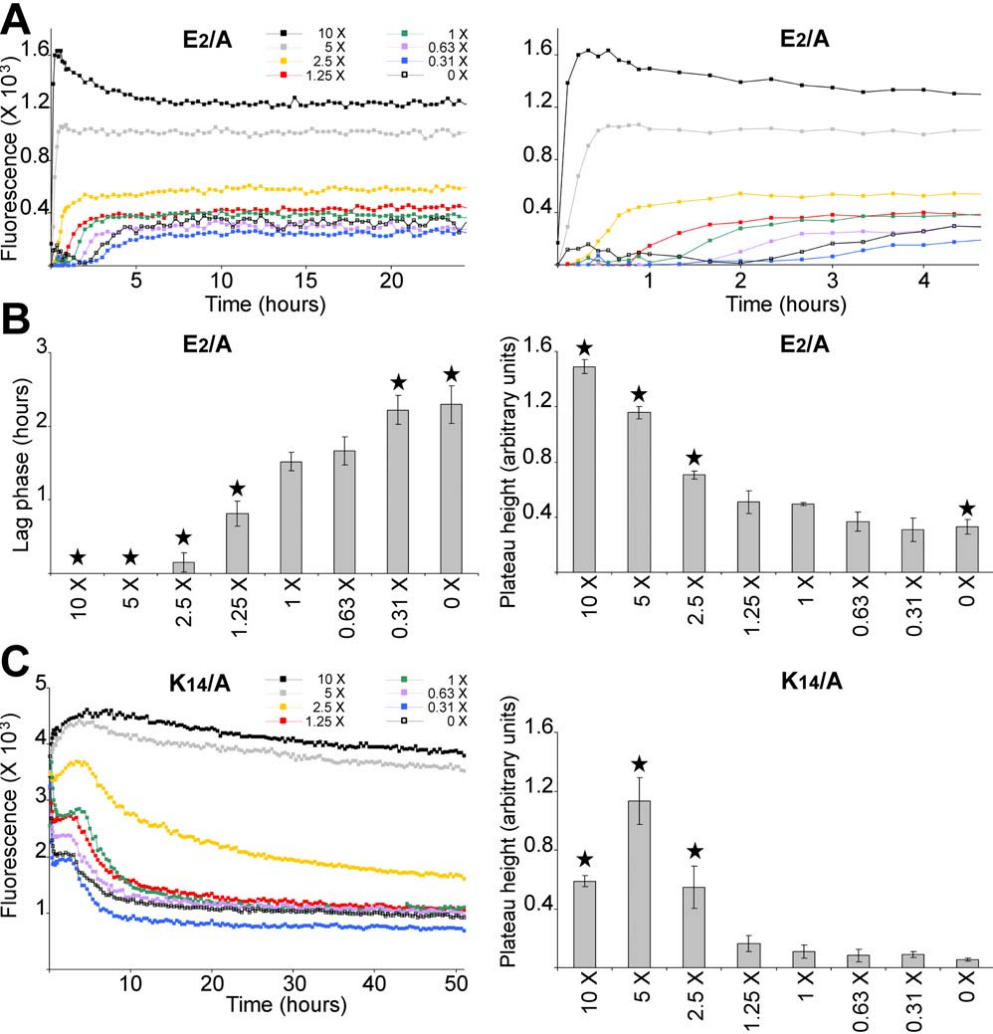
Effect of ionic strength on the fibrilization properties of mutant peptide with unstable amyloid products. Mutant peptide E_2_/A (50 µM) (A and B) or K_14_/A (100 µM) (C) were incubated with 165 µM ThT in 1.8 mM KH_2_PO_4_ and 10.1 mM NaH_2_PO_4_ with varying concentration of NaCl and KCl. The concentrations of NaCl and KCl were respectively: 0 and 0 (0 X), 42.8 mM and 0.84 mM (0.31 X), 85.6 mM and 1.7 mM (0.63 X), 136.9 mM and 2.7 mM (1 X), 171.1 mM and 3.4 mM (1.25 X), 350 mM and 6.7 mM (2.5 X), 700 mM and 13.5 mM (5 X), and 1.4 M and 27 mM (10 X). Changes in ThT fluorescence were monitored (A, with the right panel showing a scale-up of the left panel to visualize the rapid fibrilizations; and C). The lag phase of fibrilization (B, left panel) and plateau height (B, right panel; and C, right panel) are depicted. A black star signifies p<0.02 (B, left panel) and p<0.03 (B, right panel; C, right panel) when compared to 1 X NaCl and KCl.

Increasing the salt concentration, therefore the ionic strength of the solute, significantly reduced the lag phase and increased the plateau height of the E_2_/A mutant (Figure 4A and B). Similarly, the salt concentration increase was able to significantly increase the plateau height of the K_14_/A mutant, in a concentration-dependent manner (Figure 4C). Furthermore, increasing the ionic strength stabilized the assembly of the K_14_/A mutant. Collectively these data strongly suggested that an increase in ionic strength of the solute successfully shielded the uncompensated charges in either mutant. In turn, this would indicate that E_2_ and/or K_14_ were likely to be involved in electrostatic interactions, such as salt bridges.

### A lack of fibrilization does not preclude peptide interaction with AChE_586-599_

The very poor fibrilization or the absence of fibrilization of the F_3_/A, W_6_/A and M_10_/A mutants prompted us to investigate the effect of these mutants in co-fibrilization assays. Indeed if F_3_, W_6_ and M_10_ are crucial, as suggested by the CD and ThT assays (see Figures 2 and 3), for the formation of β-sheets and amyloid aggregation, it is important to determine whether the corresponding mutants could interact with the wild-type peptide AChE_586-599_ and if they could affect AChE_586-599_ fibrilization. It was previously shown that short sequences of Aβ containing Phe residues were able to bind specifically to the full length Aβ and to inhibit its fibrilization [26], [27]. Such findings could be applied to the prevention of critical interactions occurring during amyloidogenesis.

**Figure 5:**
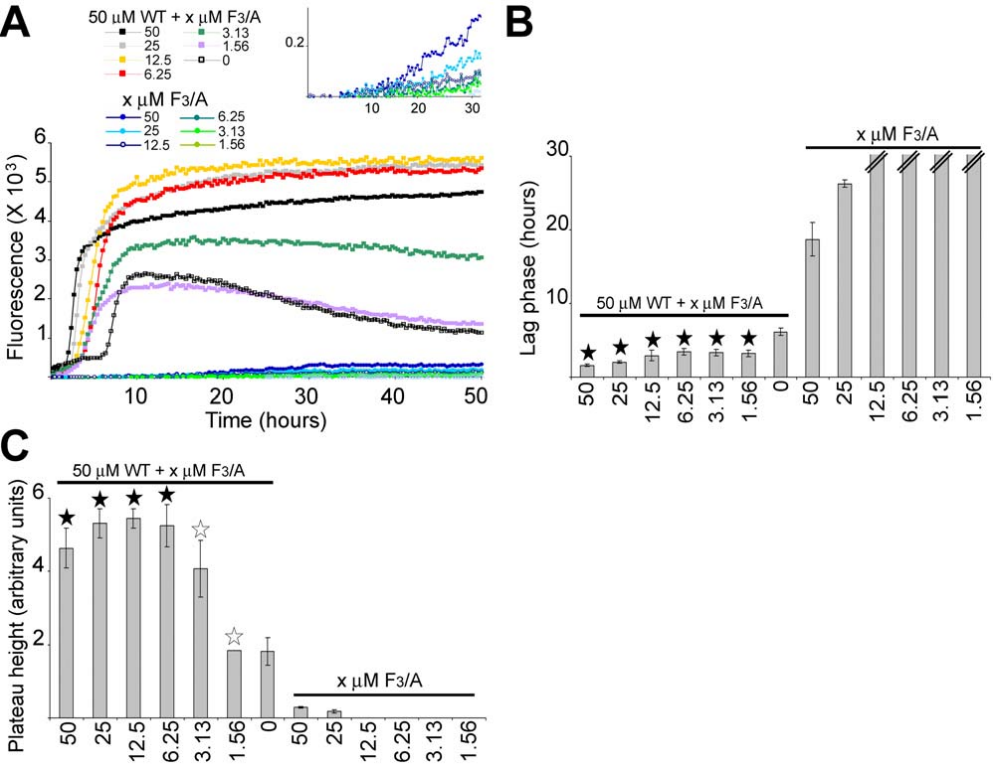
Effect of the mutant peptide F_3_/A on AChE_586-599_ fibrilization. Varying concentrations of the mutant F_3_/A were incubated with 165 µM ThT, with or without 50 µM AChE_586-599_. Changes in ThT fluorescence were monitored (A) with the lag phase of fibrilization (B) and the plateau height (C) depicted. The mutant peptide F_3_/A decreases the lag phase of AChE_586-599_ (A, with the inset showing a scale-up to visualize the fibrilization of the F_3_/A mutant on its own; and B), and increases the plateau height of AChE_586-599_ (A and C). A black star signifies p<0.03 (B) and p<0.05 (C) when compared to both 50 µM AChE_586-599_ and the equivalent concentration of the F_3_/A mutant. A white star signifies p<0.05 (C) when compared to the equivalent concentration of the F_3_/A mutant. The double bar in B indicates the absence of fibrilization (i.e. an indeterminably long lag phase).

When AChE_586-599_ (50 µM) was fibrilized in the presence of various concentrations of the F3/A mutant (ranging from 1.56 µM to equimolar), some of AChE_586-599_ fibrilization parameters were affected. First, the F_3_/A mutant was able to stabilize the final aggregation stage of AChE_586-599_ (Figure 5A). Indeed, the assembly of AChE_586-599_ on its own was not stable with the plateau reaching a maximum before decreasing (Figure 5A, black open squares). By contrast, the plateau remained stable when AChE_586-599_ was co-fibrilized with substoichiometric amounts of the F3/A mutant (from 6.25 to 50 µM). Second, the presence of the F_3_/A mutant (from 6.25 to 50 µM) also shortened the lag phase and increased the plateau height of AChE_586-599_ (p<0.005 and p<0.014 respectively) (Figure 5B and 5C). Thus, AChE_586-599_ and the F_3_/A mutant were clearly able to interact with one another.

**Figure 6:**
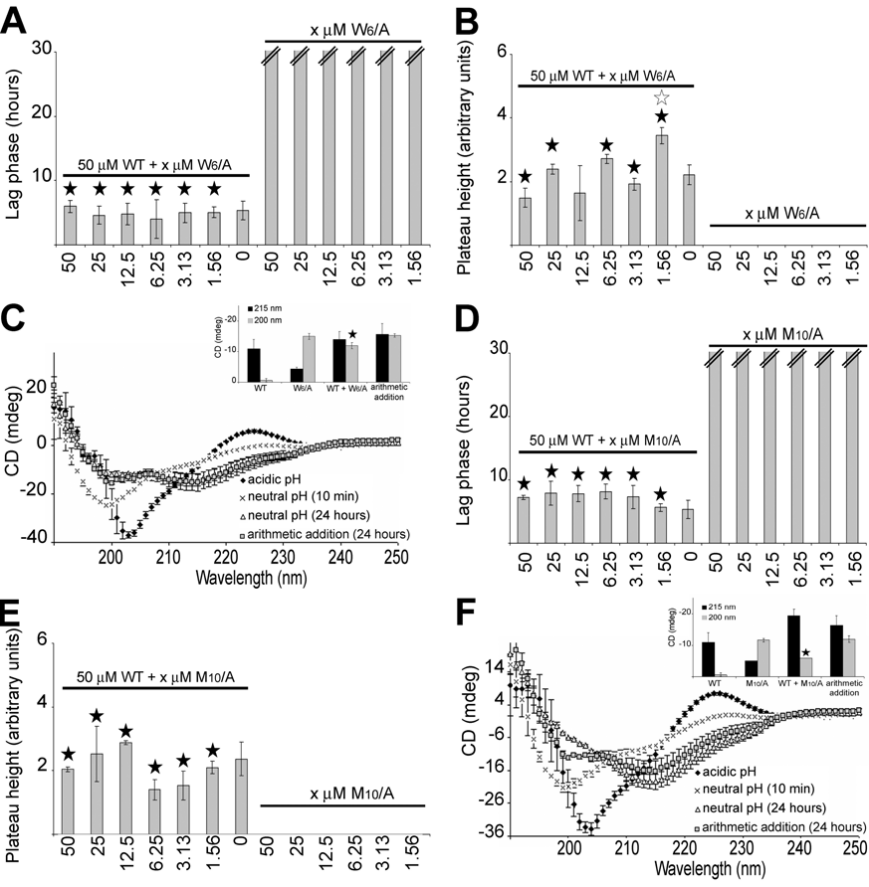
Effect of the mutant peptides W_6_/A and M_10_/A on AChE_586-599_ fibrilization. Varying concentrations of the mutant peptides were incubated with 165 µM ThT, with or without 50 µM AChE_586-599_. Changes in ThT fluorescence were measured and plotted as the lag phase of fibrilization (A and D) and the plateau height (B and E). The double bar in A and D indicates the absence of fibrilization (i.e.an indeterminably long lag phase). Far UV spectra (250 to 190 nm) before and after pH neutralization (50 mM NaH_2_PO_4_, pH 7.2) of 75 µM AChE_586-599_ with 75 µM mutant peptide (C and F). The insets show the mean residue ellipticity at 200 nm (random coil) and 215 nm (β-sheet) for 75 µM AChE_586-599_, 75 µM mutant peptide, 75 µM AChE_586-599_ with 75 µM mutant peptide, and the arithmetic addition of AChE_586-599_ to the mutant. (A and B) The mutant peptide W_6_/A does not affect either the lag phase (A, a black star signifies p<0.002 when compared to W_6_/A at the equivalent concentration) or the plateau height of AChE_586-599_ (B, a black star signifies p<0.045 when compared to W_6_/A at the equivalent concentration; a white star signifies p<0.05 when compared to AChE_586-599_). (C) The mutant peptide W_6_/A interacts with AChE_586-599_. A black star signifies p<0.014 when compared to the arithmetic addition of 75 µM W_6_/A mutant to 75 µM AChE_586-599_. (D and E) The mutant peptide M_10_/A does not affect either the lag phase (D, a black star signifies p<0.0007 when compared to M_10_/A at the equivalent concentration) or the plateau height of AChE_586-599_ (E, a black star signifies p<0.045 when compared to M_10_/A at the equivalent concentration). (F) The mutant peptide M_10_/A interacts with AChE_586-599_. A black star signifies p<0.01 when compared to the arithmetic addition of 75 µM M_10_/A mutant and 75 µM AChE_586-599_.

In contrast to the F_3_/A mutant, the W_6_/A and M_10_/A mutants did not affect AChE_586-599_ fibrilization properties. Indeed in the presence of either mutant, both the lag phase and plateau height were similar to the values for AChE_586-599_ fibrilized on its own (Figures 6A, B, D and E). However, both mutants clearly interacted with AChE_586-599_, as indicated by the far-UV CD of the mixture, which was not entirely the arithmetic addition of the two separate spectra (Figures 6 C and F). The spectra of the AChE_586-599_ and mutant mixtures after 24 hours under neutral conditions indicated the presence of both random coil (200 nm) and β-sheet (215 nm) structures. The random coil signals for the mixtures were statistically different to the signal of the arithmetic sums of AChE_586-599_ with the W_6_/A mutant or with the M_10_/A mutant (p<0.014 and p<0.01 respectively) (Figures 6C and F, insets).

### Identification of the residues important for the surfactant properties of AChE_586-599_

**Figure 7:**
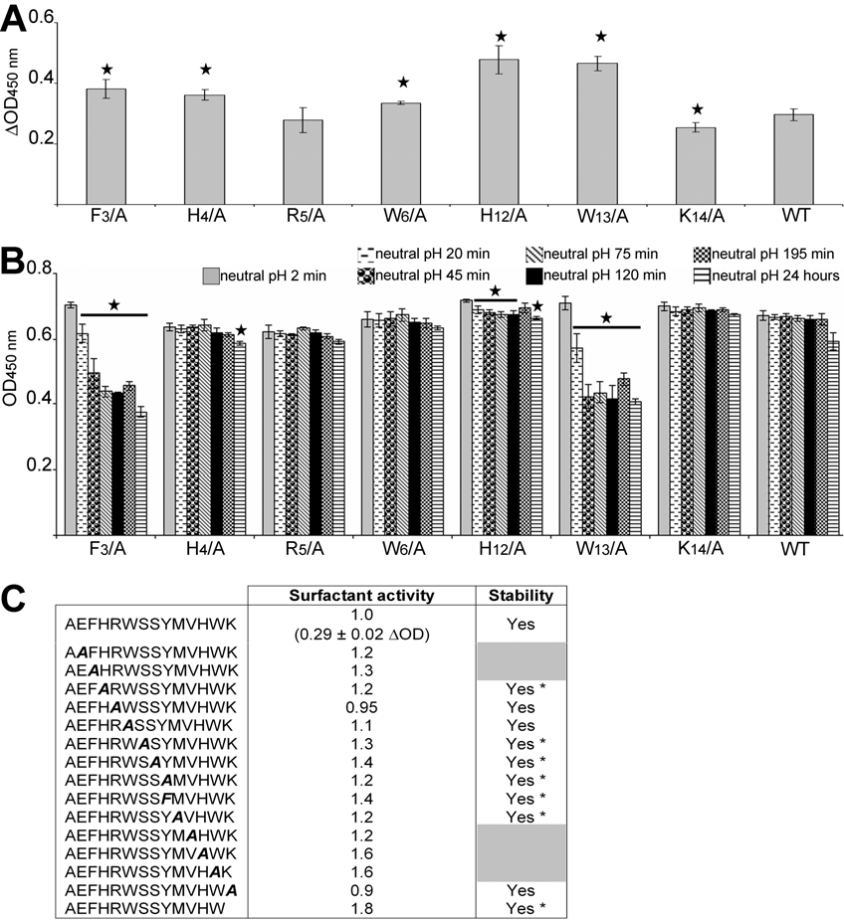
Surfactant properties of AChE_586-599_ and AChE_586-599_ mutants. Surface tension was measured before and after neutralization (1M NaH_2_PO_4_, pH 7.2). (A) Representative surfactant activity of the AChE_586-599_ mutants (50 µM). ΔOD calculations were as described in Methods. A black star signifies p<0.035 when compared to AChE_586-599_. (B) Temporal pattern of the surfactant properties for AChE_586-599_ and AChE_586-599_ mutants. The peptides shown are representatives of the different surfactant properties observed. A black star signifies p<0.05 when compared to the same peptide after 2 min at neutral pH. (C) Surfactant properties for AChE_586-599_ and all AChE_586-599_ mutants (50 µM). The properties are divided into 2 categories: surfactant activity dependent on pH (depicted by subtracting the value at acidic pH to the value at neutral pH after 2 min) and stability of the surfactant activity (indicated by stability or decay of the OD signal). The mutation within AChE_586-599_ is indicated in bold and italics. The peptides with unstable surfactant activity are indicated by grey boxes. ‘*’ indicates peptides which activity remains stable over the time course, albeit one time point. The activity for the mutant peptides is shown as fold ratio of AChE_586-599_ activity (e.g. ‘1’ represents equal value to AChE_586-599_).

Both AChE_586-599_ and Aβ possess surfactant properties. Similarly to detergents, both peptides reduce the surface tension of water by orientating their hydrophobic moiety away from the aqueous phase and ordering their amphiphilic moiety at the air-water interface [28], [29]. The surfactant activity of AChE_586-599_ was previously shown to be highly pH dependent [29]. All the substitution mutants were analyzed for surfactant activity by measuring differential absorbance, as described in Material and Methods (Figure 7). AChE_586-599_ showed a differential absorbance of 0.29±0.02 ΔOD (Figure 7A and C). Only one mutant (R_5_/A) displayed surfactant activity similar to AChE_586-599_ after 2 min neutralization and only one showed a reduced surfactant effect under these conditions (K_14_/A) (Figure 7A and C). The remaining mutants showed a larger increase of ΔOD after 2 min at neutral pH, which demonstrated that their effect on surface tension was bigger than AChE_586-599_ and also strongly pH dependent (p<0.035) (Figure 7C).

When the temporal pattern of the surfactant activity of the mutants was analyzed, it was realized that some mutants were not stably surface active (Figure 7B and C). Indeed, the surfactant activity of the E_2_/A, F_3_/A, V_11_/A, H_12_/A and W_13_/A mutants, displayed after 2 min at neutral pH, significantly decreased with time (p<0.05). This result indicates that these mutants were able to quickly segregate at the air-water interface after neutralization. The subsequent loss of surfactant effect suggests that these peptides were then either promptly leaving the air-water interface to return to the bulk, or were undergoing a conformational change at the air-water interface to reduce their effect on surface energy. By contrast, the surfactant activity of AChE_586-599_ and the other mutants was stable, which suggests that they remained stably associated with the air-water interface during the length of the time course.

### Identification of the residues important for the early steps of AChE_586-599_ oligomerization

The absence of ThT signal during the fibrilization assay did not preclude the presence of small oligomers that could be ThT negative. Therefore, we analyzed the involvement and role of the side-chains of each residue within AChE_586-599_ in oligomer formation and oligomer size distribution by performing photo-induced cross-linking of unlabeled AChE_586-599_ and AChE_586-599_ mutants (PICUP) [30]. It was previously demonstrated that the concentration of monomeric peptide during PICUP had to remain below 15 µM to avoid non-specific collision (Kenneth Baker and David J Vaux, unpublished data). In our assay, it was not possible to detect the cross-linked oligomers via conventional protein stains, presumably because the starting mass of peptide is then distributed across many individually low abundance oligomeric species. Therefore, the oligomers were detected by western-blot using the specific mouse Mab 105A, anti-AChE_586-599_ in a β-sheet conformation [23], which also provided additional information about the conformation of the cross-linked products. However, it was impossible to assay the F_3_/A, H_4_/A and R_5_/A mutants since these mutations were shown to abolish 105A immunoreactivity [23].

**Figure 8:**
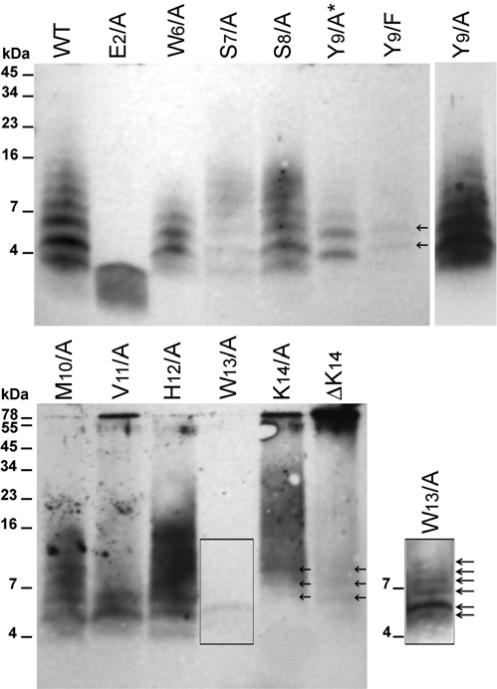
Formation of amyloid oligomers by AChE_586-599_ and AChE_586-599_ mutants. Oligomers of AChE_586-599_ and AChE_586-599_ mutants (12 µM) were cross-linked by photo-induced cross-linking. Cross-linked products were resolved (16.5% Tris-Tricine SDS-PAGE), electro-blotted onto nitrocellulose and probed with Mab 105A (specific for AChE_586-599_ in β-sheet conformation). Marker proteins are indicated. Arrows indicate low abundance oligomeric species. Due to the strength of the signal for the oligomeric species, Y_9_/A was loaded at a third of the amount of the other peptides (Y_9_/A*). The signal resulting from loading equal amount to the other peptides can be seen on the individual lane on the right hand side of the top panel (Y_9_/A). On the right hand side of the bottom panel, an overexposure of the signal for W_13_/A shows multiple oligomeric species not seen at normal exposure.

AChE_586-599_ oligomers had a distinct size distribution ranging from ∼3 to 16 kDa, with the oligomer intensity decreasing with the increase in size (Figure 8, top panel). These oligomeric forms may range from dimers to nonamers according to their observed molecular weights (AChE_586-599_ being 1.86 kDa). The most intense oligomer band was ∼5 kDa, which may correspond to trimers. Substitutions of S_7_, S_8_, Y_9_ and M_10_ to Ala yielded a qualitative distribution of oligomers similar to that of AChE_586-599_. However, the relative total amounts formed were different with S_7_/A much weaker than AChE_586-599_, M_10_/A weaker, S_8_/A similar (except the ∼5 kDa oligomers) and Y_9_/A much stronger. Therefore, the side-chains of S_7_, S_8_, Y_9_ and M_10_ were not essential for normal oligomer distribution. When Y_9_ was substituted to Phe, both the oligomer distribution and amount decreased significantly, when compared to the Y_9_/A mutant. The only oligomer bands detected were at ∼5 and 6.5 kDa, which could be trimers and tetramers. Phe differs from Tyr by missing the phenolic oxygen, which suggests that the importance of Y_9_ in the formation of oligomers bigger than tetramers may reside in the phenolic oxygen rather than the phenolic ring itself.

The substitution of W_6_ and W_13_ to Ala severely affected both the amount and size distribution of the oligomers, with the oligomers ranging from ∼3 to 9 kDa (dimers to pentamers) for W_6_/A and from ∼5 to 9 kDa (trimers to pentamers) for W_13_/A, and the oligomer amount being more reduced for W_13_/A. Thus, the side-chains of the W_6_ and W_13_ appeared to be essential and a driving force in the association into oligomers.

For the V_11_/A, K_14_/A and ΔK_14_ mutants, oligomers at ∼78 kDa (circa 42-mers) were detected. For V_11_/A and K_14_/A, the amount of these high molecular weight oligomers was reduced relative to that of the ΔK_14_ mutant. As the abundance of the band at ∼78 kDa increased, the low molecular weight oligomers were fewer and less abundant, suggesting a precursor-product relationship. Although, the high molecular weight oligomer band at ∼78 kDa was less intense for K_14_/A relative to ΔK_14_, a prominent smear of oligomers was present between ∼12 and 40 kDa. Along with the ∼78 kDa oligomers, 2 other types of oligomers ∼5 to 6.5 kDa (trimers and tetramers) were prominent for V_11_/A. In addition to the normal oligomer size distribution, substitution of H_12_ to Ala also yielded a smear of oligomers ranging from ∼12 to 20 kDa (heptamers to dodecamers). These results suggested that the side-chains of V_11_, H_12_ and K_14_ within AChE_586-599_ restrained the formation of high molecular weight oligomers and therefore controlled AChE_586-599_ oligomerization.

Mutation of E_2_ to Ala abolished the immediate formation of cross-linked oligomers larger than ∼3 kDa (dimers), suggesting that E_2_ was essential for any oligomer formation above dimers at this low concentration during this short time-course experiment.

### Morphology of the amyloid aggregates of AChE_586-599_ and AChE_586-599_ mutants

**Figure 9:**
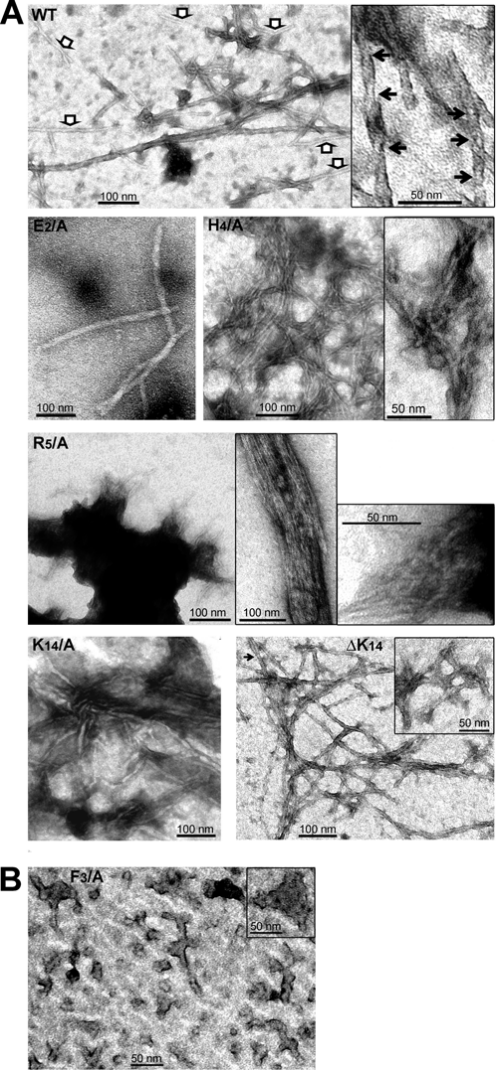
T40/IDE digestion products form amyloid protofibrils. Electron micrographs of negatively stained AChE_586-599_ and AChE_586-599_ mutants showing fibrils (A) and protofibrils (B). The white arrows in the left panel indicate thinner fibrils, and the black arrows indicate twists in the fibrils (A).

The ThT assay has allowed the determination of the aggregation potential and kinetics of the various peptides, and also provided a clue about the stability and quantity of the final amyloid products (plateau height). However, it did not give direct information about the size and morphology of the aggregates formed. Moreover, the analysis of the oligomer formation and oligomer size distribution by PICUP did not allow the study of the F_3_/A, H_4_/A and R_5_/A mutants. Thus, we used negative staining electron microscopy (EM) to examine the ultrastructure of the aggregates for the F_3_/A, H_4_/A and R_5_/A mutants and also to determine whether mutants, which were faster than AChE_586-599_ at adopting a β-sheet conformation and/or at fibrilizing (e.g. K_14_/A and ΔK_14_), could form different types of amyloid aggregates. Representative images of the morphologies observed are presented in Figure 9.

Appearance of unbranched fibrils was observed for AChE_586-599_ and mutants E_2_/A, H_4_/A, R_5_/A, K_14_/A and ΔK_14_ (Figure 9A). The fibrils formed by the mutants were different to the ones derived from the wild-type peptide. AChE_586-599_ fibrils were generally aligned, very long (>2 µm) and broad (10–15 nm) (Figure 9A, top panel) and showing typical helical twists (inset, black arrows). The periodicity of AChE_586-599_ fibrils is 26–34 nm. Some thinner (5–8 nm) and sometimes shorter fibrils were also observed for AChE_586-599_ (white arrows). By contrast, the fibrils from the E_2_/A mutant were as broad as the ones from AChE_586-599_ (∼15 nm), however they were shorter and less abundant (Figure 9A). The R_5_/A mutant formed large fibrilar aggregates, from which laterally associated (top inset) and tangled (bottom inset) fibrils were emerging (Figure 9A). Fibrils from H_4_/A mutant were abundant, thin (4–6 nm) and tangled with each other (Figure 9A), often resulting in structures resembling ‘plates’ (inset). Similarly, fibrils from ΔK_14_ mutant were more abundant, slightly thinner (8–13 nm) and more tangled than AChE_586-599_ (Figure 9A). ΔK_14_ also displayed some very short fibrils (>50 nm) (inset). The helical twists were also observed for ΔK_14_ mutant (black arrow), however this twisted morphology was less frequent than for the wild-type AChE_586-599_ fibrils. The K_14_/A mutant displayed a similar fibril morphology to the ΔK_14_ mutant, albeit the fibrils were thinner (5.5 to 8.5 nm) and fewer (Figure 9A).

Examination of F_3_/A mutant revealed predominantly spherical structures (diameter 10–16 nm), “rods” (8–13 nm wide, >50 nm long) (protofibrils) and amorphous aggregates of various sizes (some being over 50 nm wide, inset) (Figure 9B). The spherical and “rod” structures are consistent with the presence of amyloid precursors (oligomers) [31], [32], [24]. However, all of the “rods” appeared branched with the branching not following any particular dimension or orientation.

The morphology of the amyloid aggregates formed by the various peptides was consistent with the results from the fibrilization assay (e.g. the highly tangled and abundant fibrilar structures that are observed for the ΔK_14_ mutant are consistent with the very fast fibrilization kinetics determined in the ThT assay).

## Discussion

The goal of this study was to investigate the driving forces involved in the organization of a model peptide, AChE_586-599_, into β-sheet oligomers and subsequently into amyloid fibrils. The choice of AChE_586-599_ as a model peptide for amyloid assembly was guided by two criteria, which are the tractability of AChE_586-599_ assembly upon neutralization (AChE_586-599_ remains monomeric and random coil at acidic pH) and the involvement of AChE or related products in Alzheimer's disease and in the promotion of Aβ fibrilization [18], [23], [19], [20], [24]. Amyloid fibrils originating from unrelated proteins or peptides possess similar structures and morphologies [1], [5]. While overall amino acid composition is an important factor determining amyloid formation, the details of primary sequence also plays an important role. Therefore, a common view is that fibril assembly is governed by properties of the sequence backbone *and* specific side-chain interactions [7], [8], [9], [33], [10]. This would explain that a simple hydrophobic collapse would not be sufficient to drive the aggregation of any polypeptide chains under physiological conditions. On the contrary, a final ordered amyloid assembly would be determined by, and highly dependent on, a number of specific interactions between side-chains at specific positions within the sequence, which is supported by our results.

**Figure 10:**
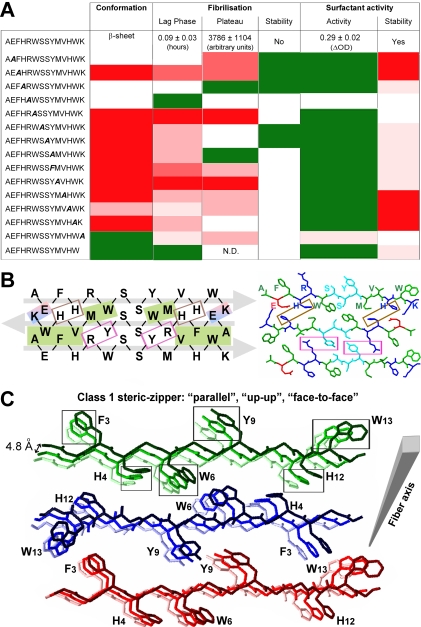
Structural model for AChE_586-599_ amyloid assembly based on conformation, fibrilization and surfactant properties of the wild type and mutant peptides. (A) Summary of the conformation, fibrilization and surfactant properties of AChE_586-599_ and mutant peptides. Mutant peptides with similar properties to AChE_586-599_ (white boxes), with enhanced properties (green boxes), with diminished properties (red boxes, with low decrease indicated by light red and strong decrease by dark red). ‘N.D.’ indicates ‘non-detectable’. (B) Model of interactions between residues of AChE_586-599_ β-strands, which form a steric-zipper interface between the fibril forming β-sheets. The steric-zipper interface is shown as an antiparallel assembly of three AChE_586-599_ β-strands. AChE_586-599_ is represented at the primary amino acid sequence level (left panel) or at the carbon backbone structure level (right panel). On the left panel, hydrophobic interactions are represented as green shaded boxes, electrostatic interactions as blue and red shaded boxes, cation-π interactions as pink boxes and potential metal binding sites as brown boxes. The grey arrows indicate the direction of the strands. On the right panel, the chains are colored by residue type with hydrophobic residues (A, F, W, M and V) in green, negatively charged (E) in red, positively charged (H, R and K) in blue, and polar (S and Y) in cyan. (C) Model of quaternary interactions within β-sheets of AChE_586-599_ within a fibril. Each β-sheet is represented with only 3 copies of AChE_586-599_ for clarity: sheet 1 colored in different shades of green, sheet 2 in different shades of blue, and sheet 3 in different shades of red. The fibril is growing from the lighter to the darker color. On the carbon backbone structure, only the side chains of aromatic residues (F_3_, H_4_, W_6_, Y_9_, H_12_ and W_13_) are represented for clarity. The boxes highlight possible aromatic interactions (π-π) between strands within a β-sheet. Within a β-sheet, AChE_586-599_ strands are stacking in a parallel arrangement. Within AChE_586-599_ fibril, the β-sheets are antiparallel, have the same sides facing each other (‘face-to-face’) and the orientation of the sheet edges facing up (‘up-up’). According to the nomenclature of Sawaya *et al*., this type of arrangement and orientation corresponds to a class 1 steric-zipper [70].

The effect of the positional scanning mutations suggested that there was a position dependence of AChE_586-599_ assembly. Indeed, some positions within the AChE_586-599_ sequence were tolerant to alanine substitutions, whereas others were restrictive. The termini of AChE_586-599_ appeared to be more permissive to mutations (e.g. E_2_, H_4_, R_5_ at the N-terminus, and H_12_ and K_14_ at the C-terminus) since they did not preclude fibrilization. By contrast, mutations within the core of AChE_586-599_ sequence were very restrictive since they sharply abolished or affected fibrilization (e.g. W_6_, S_7_, S_8_, Y_9_, M_10_ and V_11_). The only exceptions were two terminal aromatic residues, F_3_ and W_13_, which drastically affected fibrilization. Thus, one could speculate that only the positions within the AChE_586-599_ sequence providing maximal and optimal stabilizing interactions, would be affected by mutations. On one hand, the dependence of the observed amyloidogenic potentials on the position of the mutation could correlate with the β-sheet propensity and the hydrophobicity of the side-chain at this position. For example, the side-chains of A_1_ and F_3_ were optimally fitted with the side-chains of V_11_ and W_13_ to create a hydrophobic motif at the edges of the assembly (see below and the model in Figure 10B). Similarly, the side-chain of W_6_ was optimally fitted with the side-chain of M_10_ to create a hydrophobic motif at the core of the structure (see Figure 10B). It was also observed that substitutions within the core of AChE_586-599_, or modifying the hydrophobicity and/or aromaticity, affected to some extend the β-sheet propensity, with the strongest effect for F_3_, W_6_, Y_9_, M_10_, V_11_ and W_13_. Since for the H_4_/A and R_5_/A mutants, there was no perturbation of the hydrophobic patch, the stabilizing effect of A_1_-W_13_, F_3_-V_11_ and W_6_-M_10_ side-chain interactions could still occur. Moreover, substitutions of H_4_ and R_5_ did not affect the β-sheet propensity within the AChE_586-599_ sequence (see Figure 1). This could explain why the fibrilization properties of these mutants were at least as good as those of AChE_586-599_. Although substitution of E_2_ and K_14_ did not disrupt the hydrophobic patches, the mutations would abolish a putative salt bridge (as discussed below). On the other hand, previous studies have shown that during amyloid formation water molecules barely interact with the hydrophobic patches but instead cluster around the terminal charged residues [34]. This solvent effect could also contribute to the position dependence of AChE_586-599_ aggregation.

There are criteria that an amyloid structure must meet to be stable, such as the need to place charged residues outside the core amyloid structure. Moreover, electrostatic interactions, such as salt bridges, may contribute to the orientation and stability of the amyloid assembly [35], [36], [15], [37]. Furthermore, Lys and Glu residues have been often found in neighboring β-strands, and peptides rich in these two residues formed fibrils [38], [39], [36]. Massi et al. predicted that an equilibrium between electrostatic interactions and hydration determines the stability of amyloid-forming peptides, therefore that the fibrilization kinetics would be affected by pH and ionic strength [12]. We have demonstrated that by providing additional ionic strength, we successfully shielded the uncompensated charges of the E_2_/A and K_14_/A mutants resulting in a shorter lag phase for the assembly and an increase of the plateau height. For the K_14_/A mutant, the resulting aggregates were also more stable. Thus, these results suggested that specific Coulombic interactions, such as a salt bridge between E_2_ and K_14_, might occur during AChE_586-599_ oligomerization. This hypothesis is reinforced by the rapid kinetics of fibrilization and the presence of high molecular weight oligomers upon substitution of the charged K_14_ with an uncharged side-chain (Ala), which demonstrated the unequivocal involvement of the positive charge of the K_14_ side-chain. A similar effect was observed for Aβ, in which the mutation of D_23_ to Asn yielded higher oligomers, suggested to be due to the removal of the salt bridge between D_23_ and K_28_ [14]. Nilsberth et al. also found that for the Aβ arctic mutation (E_22_ to Gly), the substitution increased the rate of protofibril formation [40]. Furthermore, substitutions of K_14_, by affecting early kinetics and assembly, could destabilize the structure of the final assembly or drive the assembly into a different pathway, which would lead to different morphologies for the aggregates. In accordance with this hypothesis, the fibrils observed for the K_14_/A and ΔK_14_ mutants were shorter, and thinner and more tangled than AChE_586-599_. Similarly, the presence of an Ala instead of the N-terminal Asn, or the absence of Asn, in the peptide NFGAILSS of human islet amyloid polypeptide (IAPP) accelerated the kinetics of aggregation and modified the morphology of the fibrils, which were thinner and more tangled [41], [11]. Tenidis et al proposed that Asn directed self-assembly and lateral packing of the filaments [41].

Interestingly, the E_2_/A mutant had a stable plateau height (albeit drastically decreased) and similar lag phase of fibrilization to that of AChE_586-599_. The increase of β-sheet propensity triggered by this mutation (see Figure 1) could compensate the absence of the salt bridge with K_14_ during early assembly, which would explain the similarity of lag phase. Moreover, E_2_ was found to be essential for the formation of normal amount and normal length fibrils, which could explain the very low plateau height observed in the ThT assay for this mutant. The substitutions of E_2_ and K_14_ also altered the general properties of AChE_586-599_, such as its net charge and isoelectric point (pI). At acidic pH, the net charge of AChE_586-599_ is +4, with the side-chains of H_4_, R_5_, H_12_ and K_14_ being protonated. This high density of positive charges would prevent interactions between AChE_586-599_ molecules, which would explain the random coil conformation. By contrast, at neutral pH the net charge of AChE_586-599_ is +1, with the carboxylic group of E_2_ side-chain being deprotonated (−1), the amino group of R_5_ and K_14_ side-chains protonated (+2). By substituting E_2_ to Ala, the net charge of AChE_586-599_ became +2. By substituting R_5_ or K_14_ to Ala, the net charge of AChE_586-599_ became 0. According to Chiti et al., an increase of the net charge would trigger intermolecular repulsion, whereas a low net charge would favor aggregation [42]. Therefore, the E_2_/A mutant would be less prone to fibrilization, and the R_5_/A and K_14_/A and ΔK_14_ mutants would fibrilize more rapidly than AChE_586-599_, just as we observed. The pI of AChE_586-599_ is 8.65 and substitution decreasing it could facilitate aggregation at neutral pH. Substitution of E_2_ to Ala raises the pI to 10.00, which could explain why the E_2_/A mutant was no faster than AChE_586-599_. By contrast, substitution of R_5_ to Ala, or K_14_ to Ala or removal of K_14_ decreases the pI to 6.96. These observations are in perfect agreement with our CD and fibrilization results, in which each of these mutants was faster than AChE_586-599_ by at least one experimental measure.

Single point mutations, which were not affecting the net charge of AChE_586-599_, were found to drastically affect or to completely abolish AChE_586-599_ conformational change and fibrilization (e.g. F_3_/A and W_6_/A). Therefore, the minimization of Coulombic repulsion is not the only factor involved in amyloid assembly and interactions between side-chains, particularly of hydrophobic and aromatic nature, could provide additional energy for stabilization. The frequency of aromatic residues is low in proteins in general, however they occur very frequently in amyloid-related sequences [11], [43]. Interactions between aromatic ring planes that are parallel to each other, referred as π-π interactions or π-stackings, play a key role in molecular recognition and self-assembly, which could be the function that the aromatic residues play during amyloid formation [44], [45], [43], [46]. Moreover, aromatic residues are characterized by both a high hydrophobicity and a high β-sheet propensity. Aromatic residues are abundant in AChE_586-599_ (29% of the total residues) and we assessed their involvement by a complementary approach, including far- and near-UV CD. These assays showed that upon neutralization a conformational transition from random coil to β-sheet occurred for AChE_586-599_ and involved strong interactions between aromatic residues for the formation of tertiary or quaternary structures. Indeed, the near-UV CD clearly demonstrated that the aromatic rings had restrained mobility, as when buried, which is consistent with these residues stacking during β-sheet formation. Similar π-stacking was described for several amyloid-forming peptides, such as IAPP and Aβ [15], [25]. Additionally to the CD studies, the influence of the aromatic residues was also observed in the fibrilization and oligomerization assays. Indeed F_3_, W_6_, Y_9_ and W_13_, when mutated, appeared to significantly influence the first stage of aggregation with either no fibrilization observed or a drastically longer lag phase. This result was also confirmed by the study on oligomer formation where the F_3_/A, W_6_/A, Y_9_/F and W_13_/A mutants led to a very poor oligomerization. The effect of the substitutions on the early stage of aggregation might be related to a decrease in hydrophobicity and β-sheet propensity rather than a lack of aromatic ring, as it was proposed for other systems [47], [48]. However in later stages of the aggregation, the role of the aromatic rings of F_3_, W_6_, Y_9_ and W_13_ within AChE_586-599_ might involve π-stacking to stabilize the cross-β structure. Indeed in a number of models, the rings of Phe residues were proposed to cement together the β-strands in a β-sheet, creating a Phe zipper [49], [50], [10], [15]. Furthermore, the substitution of F_3_ or W_13_ to Ala may have affected the tight association between strands, at the paired hydrophobic patches within the steric-zipper (see green box in Figure 10B). The smaller side-chain of Ala instead of the bulky side-chain of F_3_ or W_13_ may permit increased flexibility that could have opposed an ordered assembly. Additionally to an effect on early aggregation, the F_3_ mutation to Ala resulted in spherical oligomers, short, wide and branched protofibrils, and amorphous aggregates. Similarly, a Phe to Ala substitution in the peptide NFGAILSS of human IAPP, resulted in the formation of amorphous aggregates [11]. It was previously described that the specificity and the directionality of the amyloid assembly could be provided by the specific orientation of aromatic side-chains [51], [52], [43]. Without F_3_, AChE_586-599_ may be lacking important interactions involved in the directionality of the assembly. This could result in a correct minimal assembly that failed to orientate for further linear stacking, leading to multiple branching and finally aggregation into amorphous structures. Therefore, the branched “protofibrils” and amorphous aggregates would be an amyloid dead end and no fibrils would be formed, as our EM results suggested.

The F_3_/A mutant, as mentioned above, would be less hydrophobic at the extremities of the assembly and therefore would be more exposed to the solvent. Thus, the F_3_/A mutant might not segregate at the air-water interface as stably as AChE_586-599_ could, due to its amphipathicity. By analyzing the temporal pattern of surfactant activity, we demonstrated that indeed AChE_586-599_ stably remained associated with the air-water interface, whereas the F_3_/A mutant quickly left the interface to relocate to the bulk solution. However, the F_3_/A mutant possessed a stronger surfactant activity than AChE_586-599_, after 2 min at neutral pH. This result suggested that soon after neutralization, the F_3_/A mutant was able to relocate and to aggregate faster than AChE_586-599_ at the air-water interface. This could explain the shorter lag phase observed when AChE_586-599_ was co-fibrilized with the F_3_/A mutant, as compared to AChE_586-599_ alone. Indeed if faster at aggregating, only small amount of the F_3_/A mutant would be sufficient to create nuclei to accelerate an assembly. Above a certain threshold of assembly, AChE_586-599_ aggregates would eventually relocate to the bulk solution to free the air-water interface for monomer recruitment and further assembly. Once in the bulk, AChE_586-599_ assembly would possibly be prone to dissociation or “shedding”. The F_3_/A mutant formed branched and amorphous aggregates, which by being an amyloid dead-end might be more stable than those from AChE_586-599_. This hypothesis could explain the difference of aggregate stability between the F_3_/A mutant (stable) and AChE_586-599_ (unstable), and also the fact that the F_3_/A mutant was able to stabilize AChE_586-599_ during co-fibrilization assays (e.g. by capping and slowing dissociation). Furthermore, López de la Paz et al. proposed that water molecules could act as a cement to bring strands and side-chains close enough via water-mediated hydrogen bonds, which would stabilize the amyloidogenic organization [34]. A similar effect of water molecules could stabilize the F_3_/A mutant in the bulk solution. Similarly to the F_3_/A mutant, the assembly stability at the air water-interface of the V_11_/A, H_12_/A and W_13_/A mutants was affected, which correlates with the negative effect of the substitutions on their fibrilization potential. It was previously proposed that surface tension could play an important role in the stabilization of proteins [53]. Collectively, these results suggest that AChE_586-599_ amyloid assembly could preferentially occur at an air-water interface rather than in the bulk solution, potentially due to a stabilization effect.

In addition to the non-covalent interactions described above, cation-π interactions have been also found to play a role in molecular association in biological systems [54], [55]. A cation-π interaction is a short-range electrostatic interaction between π electrons in an aromatic ring and a positively charged cation, most commonly between Arg and Tyr [56], [54]. The formation of a cation-π interaction could lower the cost of desolvating the charge of the cation and could provide a mean for burying the positively charged group within a solvent-excluding domain. Moreover, cation-π interactions are important for specificity and stability during protein association [54], [57]. In protein complexes, Arg involved in cation-π interactions were also found to be involved in cation-anion interactions, which provide long range attraction for the guanidium group and ensure the specificity of binding [54]. The importance of cation-π interaction was demonstrated for other amyloidogenic peptide [58], [59], [48]. Thus, it is possible that a cation-π interaction between R_5_ and Y_9_ occurred during AChE_586-599_ fibrilization (see Figure 10B, pink boxes), allowing the burial of the polar Arg group within the core of AChE_586-599_. Without R_5_, AChE_586-599_ rapidly formed large fibrilar aggregates composed of laterally associated and tangled fibrils, which could be due to the absence of specificity and stability provided by an R_5_-Y_9_ interaction. In addition to cation-π interaction, amino-aromatic interaction could also contribute to the formation of R_5_-Y_9_ side-chain interactions, and Y_9_ could be involved in long-range interaction with the negatively charged side-chain of E_2_ providing further specificity to the assembly. The substitution of Y_9_ with Phe, conserving only the aromatic ring, had a more deleterious effect on fibrilization than the Ala substitution and drastically impaired oligomer formation, suggesting a role of Y_9_ OH group during stacking rather than during very early assembly (formation of the nuclei).

Additionally to π-π and cation-π interactions, aromatic residues can also be involved in SH-π interactions. The Met sulfur can favorably and strongly interact with the non-polar surfaces on binding partners (specifically the aromatic face of residues), and can also engage oxygen atoms through S–O interactions and N–H groups through hydrogen bonding. Such interactions would be useful in the association between different sub-units in oligomeric proteins and could be a stabilizing force in holding two β-strands together [60]. Met has been found to preferentially associate with Trp, due to hydrogen bonding and S-aromatic interactions [61]. When M_10_ was substituted to Ala, AChE_586-599_ lost the ability to switch to a β-sheet conformation and to fibrilize. However, the M_10_/A mutant was able to form oligomers with a size distribution identical to AChE_586-599_ but less abundant, which reinforces that it is not merely the hydrophobicity of the side-chain that drives AChE_586-599_ oligomerization. It is possible that these oligomers did not bind ThT and were not able to further assemble into larger species, resulting in an absence of signal during the ThT assays. A role of M_35_ has been described in the dimerization of the Aβ protofibril [62]. Therefore, it is possible that M_10_ interacted with W_6_, through hydrophobic or S-aromatic interactions, to stabilize the AChE_586-599_ assembly. The similar effects observed for the substitutions of both W_6_ and M_10_ on fibrilization and oligomerization reinforce this hypothesis.

Another important factor able to stabilize proteins is metal chelation [63]. Studies on the binding of metal ions on amyloid proteins or peptides demonstrated that Cu(II) ions induce β-sheet formation of the unstructured amyloidogenic region of the prion protein, and Cu(II) and Zn(II) ions strongly induce Aβ fibrilization [64], [65], [66], [67], [68]. In the case of AChE_586-599_, the substitution of H_12_ to Ala affected both the fibrilization rate and the oligomerization, with a slower lag phase than AChE_586-599_, a decrease in plateau height and an unbalanced distribution of oligomers. Moreover, the substitution of H_4_ to Ala affected the morphology of the fibrils, thinner than AChE_586-599_ and tangled. Thus, it appeared that H_12_ was more “important” than H_4_, which was in agreement with the significant loss of propensity for conversion to β-strand within the sequence YMVH (the strongest propensity within AChE_586-599_), when H_12_ was mutated to Ala. A putative role for H_4_ and H_12_ in metal chelation will be the subject of further studies.

AChE_586-599_ is amphiphilic due to an alternating pattern of polar and non-polar residues, which would trigger the burial of the non-polar faces by aggregating into β-sheet structure. Although nature and evolution have disfavored such alternating pattern, sequences containing it were shown to self-assemble [8]. Polar side-chains have the advantage of forming hydrogen bonds, in addition to the van der Waals interactions. In general polar residues interact with the solvent or other polar residues. The effects of substitutions of S_7_ and S_8_ on all the AChE_586-599_ properties tested were similar and consistent. Indeed, the decrease oligomer amount for the S_7_/A and S_8_/A mutants were in agreement with the absence of fibrilization or their longer lag phases of fibrilization. The similarity and consistency upon substitution suggests that the two Ser residues might interact together rather than with other residues, creating a polar-polar interaction through hydrogen bonding, which would be in agreement with the strong correlation found between Ser-Ser pairing in β-sheets [39].

**Figure 11:**
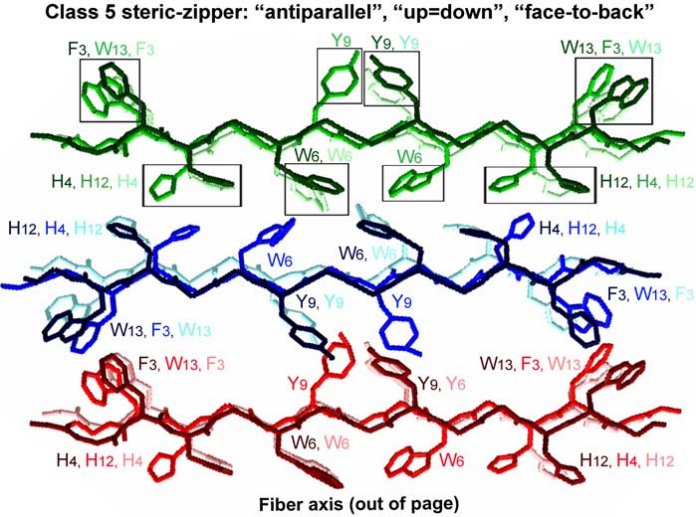
Model of quaternary interactions within β-sheets of AChE_586-599_ within a fibril of class 5. Each β-sheet is represented with only 3 copies of AChE_586-599_ for clarity: sheet 1 colored in different shades of green, sheet 2 in different shades of blue, and sheet 3 in different shades of red. On the carbon backbone structure, only the side chains of aromatic residues (F_3_, H_4_, W_6_, Y_9_, H_12_ and W_13_) are represented for clarity. The boxes highlight possible aromatic interactions (π-π) between strands within a β-sheet. Within a β-sheet, AChE_586-599_ strands are stacking in an antiparallel arrangement. Within AChE_586-599_ fibril, the β-sheets are antiparallel, have the same sides facing each other (‘face-to-face’), however the orientation of the strand edges within a sheet alternate between up and down (‘up = down’). According to the nomenclature of Sawaya *et al*., this type of arrangement and orientation corresponds to a class 5 steric-zipper [70].

It is thought that extended parallel β-sheets are less stable than antiparallel ones [69], [34]. This is based on the fact that in antiparallel β-sheets, most contacts along the fibril axis are between non-identical and complementary residues, which allow more variability in the hydrogen bonds and side-chain interactions, and also in the geometry of the interactions. This variability would allow a greater number of possible conformations and alignments for the strands. By contrast, in a parallel arrangement, the contacts are in between identical residues and the optimal geometry of the hydrogen bonding would therefore be linear. The presence of uncompensated opposite charges on each peptide plays a fundamental role in favoring an arrangement in which the distance between identical charges is maximized. In the case of AChE_586-599_, this would favor an antiparallel organization to avoid high electrostatic repulsion due to E_2_ and K_14_. This is a conclusion already tentatively suggested by ELISA on these mutants, using the MAb 105A [23]. A good agreement and correlation were found between all the assays and properties for AChE_586-599_ aggregation, and are summarized in Figure 10A. In accord with the experimental observations, and taking all the previous arguments and interactions into consideration, we attempted to model the assembly of AChE_586-599_, in which we have considered only an antiparallel arrangement for the β-sheets (Figure 10B and C). Our model fits with the position dependence of assembly (restrictive and permissive positions for substitutions) and the putative interactions described above. Indeed, figure 10B shows an electrostatic interaction between E_2_ and K_14_ (blue and red shaded box); two hydrophobic patches, the first one at the edges of the assembly (A_1_-W_13_ and F_3_-V_11_) and the second within the core of the assembly (W_6_-M_10_, which could also include an S-aromatic interaction) (green shaded boxes); cation-π interactions between R_5_ and Y_9_ (pink boxes); putative site for metal chelation between H_4_ and H_12_ (brown boxes); and polar-polar interaction between S_7_ and S_8_ (non boxed). All these interactions fit with the recent proposal of a steric-zipper forming the basic surface from which the β-sheet stacking occurs and the fibril elongates [70]. Figure 10C represents the formation of the β-sheets along the fiber axis and the putative quaternary interactions stabilizing and reinforcing such assembly. Such interactions during stacking of the strands within the β-sheet would be π-π between aromatic rings (Phe, Tyr and Trp residues) and metal chelation (His and Tyr residues). Our experiments were not able to ascertain the orientation of the strand edges (both edges ‘up’, or one ‘up’ and one ‘down’,) or of the strand faces (face-to-face or face-to-back, with the same or different faces adjacent to one another) during the stacking. The differences between the types of orientation would see the assignment to different classes of steric-zipper, according to the nomenclature of Sawaya et al., and the identity of the aromatic residues involved in the π-π interactions [70]. Class 2, 3, 6, 7 and 8 of steric zippers were ruled out due to the parallel arrangement of their β-sheets. We found that optimal π-π stacking, as represented and highlighted on Figure 10C, could be achieved only when AChE_586-599_ β-sheets have the same sides facing each other (‘face-to-face’) and the orientation of the sheet edges facing up (‘up-up’). According to the nomenclature of Sawaya et al., this type of arrangement and orientation corresponds to a class 1 steric-zipper [70]. The strands within a β-sheet are stacking in a parallel arrangement, whereas the β-sheets are antiparallel. We noted that the only other class with similar side-chain interaction within the steric-zipper, class 5 (the strands within a β-sheet are stacking in an antiparallel arrangement; the β-sheets are antiparallel, and have the same sides facing each other, ‘face-to-face’), led to a model with poor π-π stacking (Figure 11). However, the orientation of the stacking remains to be determined.

In summary, the analysis of stabilizing or destabilizing effects of residue substitutions on the amyloid assembly of AChE_586-599_ has provided evidence for the critical role of specific side-chain interactions in the stabilization of nascent aggregates and for the position dependence of these side-chains upon polymerization and fibril formation. Systematic dissections of the critical residues and interactions driving amyloid assembly, and of the chemical details underlying the molecular recognition process could provide invaluable information on such a poorly understood and complicated process. The benefits of such an understanding could be applied to the wider field of protein folding since an increasing number of non-pathogenic polypeptides have been shown to form amyloid fibrils under certain conditions [71], [72], [73], [74]. Another important application would be in the biological and medical fields by helping in the design of synthetic molecules to prevent the critical interactions occurring during amyloidogenesis (e.g. capping peptides abolishing Aβ fibrilization and blocking of π-stacking interactions) [26], [27].

## Materials and Methods

### Synthetic peptides and antibodies

AChE_586-599_ and AChE_586-599_ mutants were prepared as described [23]. Specific mouse Mab (105A) anti-AChE_586-599_ in a β-sheet conformation was previously described [23].

### Preparation of amyloid oligomers

AChE_586-599_ and AChE_586-599_ mutants oligomers (12 µM) were covalently cross-linked by photo-activation using the photo-induced cross-linking of unlabelled proteins (PICUP) as previously described [24]. However, the light was filtered through a 450 nm UV filter and the reaction mixture was exposed to 5 flashes of light (xenon lamp).

### SDS-PAGE and Western-blot

Amyloid oligomers were resolved on 10% Tris-Tricine SDS-PAGE and electro-blotted onto nitrocellulose. Nitrocellulose membranes were blocked with 5% (w/v) non-fat milk in PBS and incubated with the Mab 105A recognizing AChE_586-599_ in β-sheet conformation, followed by anti-mouse IgG conjugated to horseradish peroxidase (HRP). Products were visualized by enhanced chemiluminescence.

### Circular dichroism

CD-spectra were recorded from 250 to 190 nm (far-UV) and from 320 to 240 nm (near-UV) at 20°C in a quartz cuvette (1 mm path length) using a Jasco J-720 spectropolarimeter. The mean spectra of multiple scans (scan speed of 50 nm min^−1^ and response time 4 sec) were collected. The spectra were blank subtracted and normalized to molar ellipticity. At least three independent assays were performed and analyzed with the two-sample Student's t-test.

### Fibrilization experiments

The assays were performed in a 96-well plate (black wall, clear bottom; Greiner, UK) with 165 µM ThT in PBS. ThT fluorescence (excitation 450 nm, emission 480 nm) was measured at 37°C every 6 min, with 5 min shaking after every measurement, on a BMG Polarstar plate reader. The values of buffer-ThT were subtracted from the values of peptide-ThT. At least three independent assays were performed and analyzed with the two-sample Student's t-test.

To investigate the effect of ionic strength, the fibrilization experiment were carried as described above except that the peptides were incubated with 165 µM ThT in 1.8 mM KH_2_PO_4_ and 10.1 mM NaH_2_PO_4_ with varying concentration of NaCl (from 0 to 1.4 M) and KCl (from 0 to 27 mM).

### Surface tension measurement

Analyses were performed in a 96-well plate format, as described [29], [24]. Briefly, AChE_586-599_ and AChE_586-599_ mutants were re-suspended in 80 µL 200 mM sodium acetate pH 3 and surface tension measured at 450 nm (BMG Polarstar plate reader) before and at various time points after neutralization (20 µL 1M NaH_2_PO_4_, pH7.2). ΔOD = (OD_offset position_–OD_central position_)_neutral pH 2min_–(OD_offset position_–OD_central position_)_acidic pH_. At least three independent assays were performed and analyzed with the two-sample Student's t-test.

### Electron microscopy

200 µM AChE_586-599_ and AChE_586-599_ mutants in 50 mM NaH_2_PO_4_ pH 7.2 were incubated for 36 hours. Then the samples were adsorbed onto Formvar-coated 400 mesh copper grids, air dried, washed with distilled water, negatively stained with 2% aqueous uranyl acetate and viewed with a Zeiss Omega 912 microscope.

### Structural Model Building

The fibrillar model was built with the DeepView program [75]. Starting with one β-strand aligned along the x-axis, a second β-strand, which is a copy of the first strand rotated by 180° along the z-axis, was placed next to the first strand along the y-axis separated by 10 Å. A third strand, an exact copy of the first strand is placed next to the second strand along the y-axis, again separated by 10 Å. Adjustment was made to the second and third strands to ensure that the side-chains on separate strands are intercalating with each other. These three strands constitute a layer or steric-zipper. For Class 1 fibril, the second and third layers were added by translating the original layer by 4.8 Å and 9.6 Å along the z-axis. For the Class 5 fibrils, the second layer was obtained by rotating the original layer by 180° along the y-axis and then by translation so that it laid on top of the original layer along the z-axis with a separation of 4.8 Å. The third layer was a translation of the first layer by 9.6 Å along the z-axis. Adjustments were made to the second and third layers to allow for the correct configuration for hydrogen bonding. Computations for energy minimization were done *in vacuo* using the GROMOS96 43B1 parameter set without reaction field, as implemented within Swiss-PdbViewer.

## References

[1] Harper JD, Lansbury PT (1997). Models of amyloid seeding in Alzheimer's disease and scrapie: mechanistic truths and physiological consequences of the time-dependent solubility of amyloid proteins.. Annu Rev Biochem.

[2] Westermark P (2005). Aspects on human amyloid forms and their fibril polypeptides.. Febs J.

[3] Klunk WE, Pettegrew JW, Abraham DJ (1989). Quantitative evaluation of congo red binding to amyloid-like proteins with a beta-pleated sheet conformation.. J Histochem Cytochem.

[4] LeVine H (1993). Thioflavine T interaction with synthetic Alzheimer's disease beta-amyloid peptides: detection of amyloid aggregation in solution.. Protein Sci.

[5] Rochet JC, Lansbury PT (2000). Amyloid fibrillogenesis: themes and variations.. Curr Opin Struct Biol.

[6] Sipe JD, Cohen AS (2000). Review: history of the amyloid fibril.. J Struct Biol.

[7] Smith CK, Regan L (1995). Guidelines for protein design: the energetics of beta sheet side chain interactions.. Science.

[8] West MW, Wang W, Patterson J, Mancias JD, Beasley JR (1999). De novo amyloid proteins from designed combinatorial libraries.. Proc Natl Acad Sci U S A.

[9] Klimov DK, Thirumalai D (2003). Dissecting the assembly of Abeta16-22 amyloid peptides into antiparallel beta sheets.. Structure.

[10] Zanuy D, Haspel N, Tsai HH, Ma B, Gunasekaran K (2004). Side chain interactions determine the amyloid organization: a single layer beta-sheet molecular structure of the calcitonin peptide segment 15-19.. Phys Biol.

[11] Azriel R, Gazit E (2001). Analysis of the minimal amyloid-forming fragment of the islet amyloid polypeptide. An experimental support for the key role of the phenylalanine residue in amyloid formation.. J Biol Chem.

[12] Massi F, Klimov D, Thirumalai D, Straub JE (2002). Charge states rather than propensity for beta-structure determine enhanced fibrillogenesis in wild-type Alzheimer's beta-amyloid peptide compared to E22Q Dutch mutant.. Protein Sci.

[13] Reches M, Porat Y, Gazit E (2002). Amyloid fibril formation by pentapeptide and tetrapeptide fragments of human calcitonin.. J Biol Chem.

[14] Bitan G, Vollers SS, Teplow DB (2003). Elucidation of primary structure elements controlling early amyloid beta-protein oligomerization.. J Biol Chem.

[15] Makin OS, Atkins E, Sikorski P, Johansson J, Serpell LC (2005). Molecular basis for amyloid fibril formation and stability.. Proc Natl Acad Sci U S A.

[16] Haass C, Selkoe DJ (2007). Soluble protein oligomers in neurodegeneration: lessons from the Alzheimer's amyloid beta-peptide.. Nat Rev Mol Cell Biol.

[17] Atwood CS, Martins RN, Smith MA, Perry G (2002). Senile plaque composition and posttranslational modification of amyloid-beta peptide and associated proteins.. Peptides.

[18] Saez-Valero J, Sberna G, McLean CA, Small DH (1999). Molecular isoform distribution and glycosylation of acetylcholinesterase are altered in brain and cerebrospinal fluid of patients with Alzheimer's disease.. J Neurochem.

[19] Rees TM, Berson A, Sklan EH, Younkin L, Younkin S (2005). Memory deficits correlating with acetylcholinesterase splice shift and amyloid burden in doubly transgenic mice.. Curr Alzheimer Res.

[20] Diamant S, Podoly E, Friedler A, Ligumsky H, Livnah O (2006). Butyrylcholinesterase attenuates amyloid fibril formation in vitro.. Proc Natl Acad Sci U S A.

[21] Cottingham MG, Hollinshead MS, Vaux DJ (2002). Amyloid fibril formation by a synthetic peptide from a region of human acetylcholinesterase that is homologous to the Alzheimer's amyloid-beta peptide.. Biochemistry.

[22] Yoon S, Welsh WJ (2004). Detecting hidden sequence propensity for amyloid fibril formation.. Protein Sci.

[23] Cottingham MG, Voskuil JL, Vaux DJ (2003). The intact human acetylcholinesterase C-terminal oligomerization domain is alpha-helical in situ and in isolation, but a shorter fragment forms beta-sheet-rich amyloid fibrils and protofibrillar oligomers.. Biochemistry.

[24] Jean L, Thomas B, Tahiri-Alaoui A, Shaw M, Vaux DJ (2007). Heterologous amyloid seeding: revisiting the role of acetylcholinesterase in Alzheimer's disease.. PLoS ONE.

[25] Jack E, Newsome M, Stockley PG, Radford SE, Middleton DA (2006). The organization of aromatic side groups in an amyloid fibril probed by solid-state 2H and 19F NMR spectroscopy.. J Am Chem Soc.

[26] Tjernberg LO, Naslund J, Lindqvist F, Johansson J, Karlstrom AR (1996). Arrest of beta-amyloid fibril formation by a pentapeptide ligand.. J Biol Chem.

[27] Kuner P, Bohrmann B, Tjernberg LO, Naslund J, Huber G (2000). Controlling polymerization of beta-amyloid and prion-derived peptides with synthetic small molecule ligands.. J Biol Chem.

[28] Soreghan B, Kosmoski J, Glabe C (1994). Surfactant properties of Alzheimer's A beta peptides and the mechanism of amyloid aggregation.. J Biol Chem.

[29] Cottingham MG, Bain CD, Vaux DJ (2004). Rapid method for measurement of surface tension in multiwell plates.. Lab Invest.

[30] Fancy DA, Kodadek T (1999). Chemistry for the analysis of protein-protein interactions: rapid and efficient cross-linking triggered by long wavelength light.. Proc Natl Acad Sci U S A.

[31] Seilheimer B, Bohrmann B, Bondolfi L, Muller F, Stuber D (1997). The toxicity of the Alzheimer's beta-amyloid peptide correlates with a distinct fiber morphology.. J Struct Biol.

[32] Lashuel HA, Petre BM, Wall J, Simon M, Nowak RJ (2002). Alpha-synuclein, especially the Parkinson's disease-associated mutants, forms pore-like annular and tubular protofibrils.. J Mol Biol.

[33] Lopez de la Paz M, Serrano L (2004). Sequence determinants of amyloid fibril formation.. Proc Natl Acad Sci U S A.

[34] Lopez de la Paz M, de Mori GM, Serrano L, Colombo G (2005). Sequence dependence of amyloid fibril formation: insights from molecular dynamics simulations.. J Mol Biol.

[35] Petkova AT, Ishii Y, Balbach JJ, Antzutkin ON, Leapman RD (2002). A structural model for Alzheimer's beta -amyloid fibrils based on experimental constraints from solid state NMR.. Proc Natl Acad Sci U S A.

[36] Tjernberg L, Hosia W, Bark N, Thyberg J, Johansson J (2002). Charge attraction and beta propensity are necessary for amyloid fibril formation from tetrapeptides.. J Biol Chem.

[37] Tarus B, Straub JE, Thirumalai D (2005). Probing the initial stage of aggregation of the Abeta(10-35)-protein: assessing the propensity for peptide dimerization.. J Mol Biol.

[38] Zhang S, Holmes T, Lockshin C, Rich A (1993). Spontaneous assembly of a self-complementary oligopeptide to form a stable macroscopic membrane.. Proc Natl Acad Sci U S A.

[39] Wouters MA, Curmi PM (1995). An analysis of side chain interactions and pair correlations within antiparallel beta-sheets: the differences between backbone hydrogen-bonded and non-hydrogen-bonded residue pairs.. Proteins.

[40] Nilsberth C, Westlind-Danielsson A, Eckman CB, Condron MM, Axelman K (2001). The ‘Arctic’ APP mutation (E693G) causes Alzheimer's disease by enhanced Abeta protofibril formation.. Nat Neurosci.

[41] Tenidis K, Waldner M, Bernhagen J, Fischle W, Bergmann M (2000). Identification of a penta- and hexapeptide of islet amyloid polypeptide (IAPP) with amyloidogenic and cytotoxic properties.. J Mol Biol.

[42] Chiti F, Stefani M, Taddei N, Ramponi G, Dobson CM (2003). Rationalization of the effects of mutations on peptide and protein aggregation rates.. Nature.

[43] Gazit E (2002). A possible role for pi-stacking in the self-assembly of amyloid fibrils.. Faseb J.

[44] Claessens CG, Stoddart JF (1997). p-p interactions in self-assembly.. J Phys Org Chem.

[45] Gillard RE, Raymo FM, Stoddart JF (1997). Controlling self-assembly.. Chem Eur J.

[46] Waters ML (2002). Aromatic interactions in model systems.. Curr Opin Chem Biol.

[47] Wu C, Lei H, Duan Y (2005). The role of Phe in the formation of well-ordered oligomers of amyloidogenic hexapeptide (NFGAIL) observed in molecular dynamics simulations with explicit solvent.. Biophys J.

[48] Bemporad F, Taddei N, Stefani M, Chiti F (2006). Assessing the role of aromatic residues in the amyloid aggregation of human muscle acylphosphatase.. Protein Sci.

[49] Sikorski P, Atkins ED, Serpell LC (2003). Structure and texture of fibrous crystals formed by Alzheimer's abeta(11-25) peptide fragment.. Structure.

[50] Naito A, Kamihira M, Inoue R, Saito H (2004). Structural diversity of amyloid fibril formed in human calcitonin as revealed by site-directed 13C solid-state NMR spectroscopy.. Magn Reson Chem.

[51] Shetty AS, Zhang J, Moore JS (1996). Aromatic -p Stacking in Solution as Revealed through the Aggregation of Phenylacetylene Macrocycles.. J Am Chem Soc.

[52] McGaughey GB, Gagne M, Rappe AK (1998). pi-Stacking interactions. Alive and well in proteins.. J Biol Chem.

[53] Lin TY, Timasheff SN (1996). On the role of surface tension in the stabilization of globular proteins.. Protein Sci.

[54] Crowley PB, Golovin A (2005). Cation-pi interactions in protein-protein interfaces.. Proteins.

[55] Paddock ML, Weber KH, Chang C, Okamura MY (2005). Interactions between cytochrome c2 and the photosynthetic reaction center from Rhodobacter sphaeroides: the cation-pi interaction.. Biochemistry.

[56] Ma JC, Dougherty DA (1997). The Cationminus signpi Interaction.. Chem Rev.

[57] Chakkaravarthi S, Gromiha MM (2006). Analysis of cation π interactions to the structural stability of RNA binding proteins.. Polymer.

[58] Yoshida H, Matsushima N, Kumaki Y, Nakata M, Hikichi K (2000). NMR studies of model peptides of PHGGGWGQ repeats within the N-terminus of prion proteins: a loop conformation with histidine and tryptophan in close proximity.. J Biochem (Tokyo).

[59] Zahn R (2003). The octapeptide repeats in mammalian prion protein constitute a pH-dependent folding and aggregation site.. J Mol Biol.

[60] Pal D, Chakrabarti P (2001). Non-hydrogen bond interactions involving the methionine sulfur atom.. J Biomol Struct Dyn.

[61] Samanta U, Pal D, Chakrabarti P (2000). Environment of tryptophan side chains in proteins.. Proteins.

[62] Petkova AT, Yau WM, Tycko R (2006). Experimental constraints on quaternary structure in Alzheimer's beta-amyloid fibrils.. Biochemistry.

[63] Kellis JT, Todd RJ, Arnold FH (1991). Protein stabilization by engineered metal chelation.. Biotechnology (N Y).

[64] Atwood CS, Moir RD, Huang X, Scarpa RC, Bacarra NM (1998). Dramatic aggregation of Alzheimer abeta by Cu(II) is induced by conditions representing physiological acidosis.. J Biol Chem.

[65] Liu ST, Howlett G, Barrow CJ (1999). Histidine-13 is a crucial residue in the zinc ion-induced aggregation of the A beta peptide of Alzheimer's disease.. Biochemistry.

[66] Miura T, Suzuki K, Kohata N, Takeuchi H (2000). Metal binding modes of Alzheimer's amyloid beta-peptide in insoluble aggregates and soluble complexes.. Biochemistry.

[67] Yang DS, McLaurin J, Qin K, Westaway D, Fraser PE (2000). Examining the zinc binding site of the amyloid-beta peptide.. Eur J Biochem.

[68] Jones CE, Abdelraheim SR, Brown DR, Viles JH (2004). Preferential Cu2+ coordination by His96 and His111 induces beta-sheet formation in the unstructured amyloidogenic region of the prion protein.. J Biol Chem.

[69] Salemme FR, Weatherford DW (1981). Conformational and geometrical properties of beta-sheets in proteins. I. Parallel beta-sheets.. J Mol Biol.

[70] Sawaya MR, Sambashivan S, Nelson R, Ivanova MI, Sievers SA (2007). Atomic structures of amyloid cross-beta spines reveal varied steric zippers.. Nature.

[71] Guijarro JI, Sunde M, Jones JA, Campbell ID, Dobson CM (1998). Amyloid fibril formation by an SH3 domain.. Proc Natl Acad Sci U S A.

[72] Fandrich M, Fletcher MA, Dobson CM (2001). Amyloid fibrils from muscle myoglobin.. Nature.

[73] Stefani M, Dobson CM (2003). Protein aggregation and aggregate toxicity: new insights into protein folding, misfolding diseases and biological evolution.. J Mol Med.

[74] Uversky VN, Fink AL (2004). Conformational constraints for amyloid fibrillation: the importance of being unfolded.. Biochim Biophys Acta.

[75] Guex N, Peitsch MC (1997). SWISS-MODEL and the Swiss-PdbViewer: an environment for comparative protein modeling.. Electrophoresis.

